# Bubble ascent and rupture in mud volcanoes

**DOI:** 10.1098/rsos.231555

**Published:** 2024-07-31

**Authors:** Maxwell L. Rudolph, Kirti Chandra Sahu, Nikos Savva, András Szilágyi, Zoltán Hórvölgyi, Péter Márton, Ádám Tajti, Károly Szép, Boglárka Balog, Manoj Kumar Tripathi, Harishankar Manikantan, Ferenc L. Forray, Michael Manga, Peter Hantz

**Affiliations:** ^1^ Department of Earth and Planetary Sciences, University of California, Davis, CA, USA; ^2^ Department of Chemical Engineering, Indian Institute of Technology Hyderabad, Hyderabad, India; ^3^ Department of Mathematics and Statistics, University of Cyprus, Nicosia, Cyprus; ^4^ The Cyprus Institute, Computation‐based Science and Technology Research Center, Nicosia, Cyprus; ^5^ Department of Physical Chemistry and Materials Science, Faculty of Chemical Technology and Biotechnology, Budapest University of Technology and Economics, Budapest, Hungary; ^6^ Bálint Analitika Kft., Budapest, Hungary; ^7^ University of Pannonia, Veszprém, Hungary; ^8^ Renovatív Attitűd Kft, Boldogkőváralja, Hungary; ^9^ Indian Institute of Science Education and Research, Bhopal, Madhya Pradesh, India; ^10^ Department of Chemical Engineering, University of California, Davis, CA, USA; ^11^ Department of Geology, Babeș-Bolyai University, Cluj / Kolozsvár, Romania; ^12^ Department of Earth and Planetary Science, University of California, Berkeley, CA, USA; ^13^ Department of Organic Chemistry, Eötvös Loránd University, Budapest, Hungary; ^14^ Fibervar LLC., Cluj / Kolozsvár, Romania

**Keywords:** rupture, bubble, rheology, mud volcano, fragmentation

## Abstract

Large gas bubbles can reach the surface of pools of mud and lava where they burst, often through the formation and expansion of circular holes. Bursting bubbles release volatiles and generate spatter, and hence play a key role in volcanic degassing and volcanic edifice construction. Here, we study the ascent and rupture of bubbles using a combination of field observations at Pâclele Mici (Romania), laboratory experiments with mud from the Imperial Valley (California, USA), numerical simulations and theoretical models. Numerical simulations predict that bubbles ascend through the mud as elliptical caps that develop a dimple at the apex as they impinge on the free surface. We documented the rupture of bubbles in nature and under laboratory conditions using high-speed video. The bursting of mud bubbles starts with the nucleation of multiple holes, which form at a near-constant rate and in quick succession. The quasi-circular holes rapidly grow and coalesce, and the sheet evolves towards a filamentous structure that finally falls back into the mud pool, sometimes breaking up into droplets. The rate of expansion of holes in the sheet can be explained by a generalization of the Taylor–Culick theory, which is shown to hold independent of the fluid rheology.

## 1. Introduction

**Figure 1.**
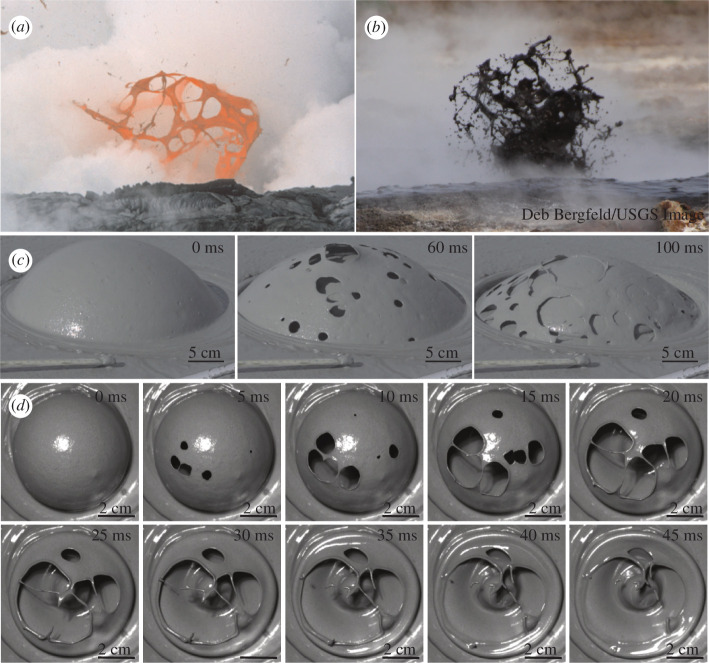
Lava and mud bubbles with holes, in igneous and mud volcanoes, respectively. (*a*) Kīlauea, Hawaii 1988 (United States Geological Survey (USGS), Public Domain Images), (*b*) Imperial Valley, California (Deb Bergfeld/USGS, Public Domain). (*c*) Formation and rupture of a mud bubble at Pâclele Mici, Buzău, Romania. (*d*) The sequence of images shows a bubble bursting in a laboratory experiment (experiment E6) using mud from the Imperial Valley, California, USA.

Gases that exsolve and expand in erupting mud or magma can form large bubbles that ascend to and burst at the surface of mud pools, lava lakes and lava flows (figure 1). When bubbles reach the surface of these non-Newtonian, shear-thinning fluids, they initially form hemispheric structures in which the gas is enveloped by a thin fluid sheet of mud or lava. The subsequent dynamics of the bubbles often lead to an interesting sequence of events (figure 1). As a gas bubble expands, rupture occurs at multiple sites within the thinning fluid sheet. This phenomenon is surprising because bubbles in Newtonian fluids often rupture when a single hole forms at the apex of the bubble (e.g. [1]). In bursting mud bubbles, the holes rapidly expand, and the fluid sheet falls owing to the loss of pressure. In the meantime, the sheet undergoes topological changes and evolves towards a web-like structure that may fragment into droplets, some of which may be ejected (e.g. [2]). A multitude of phenomena involving thinning liquid sheets display similar nucleation and hole growth dynamics (e.g. [3]), including planar water jets, films of melted iron [4], mercury sheets [5], expanding tin microdroplets [6] and lava structures [7]. Therefore, exploring the details of bubble dynamics in mud can serve as an analogue for bubbles in magma [7], and can contribute to a better understanding of various phenomena in interface science (e.g. [8]).

We present new observations and models of bubble ascent, expansion and rupture at natural mud volcanoes and under laboratory conditions. Depending on water content, mud can behave as either a fluid or a solid. Gas ascends by viscous flow at high water contents and by fracture at lower water contents (e.g. [9,10]). In the field and laboratory conditions discussed herein, mud is always in the fluid regime. We first describe the two sites used for field observations and sample collection. We characterize the rheology of the mud in which bubbles burst at each location.The ascent of bubbles through the mud as they approach a free surface is modelled numerically by solving the Navier–Stokes equations with a rheological model informed by our laboratory measurements. We confirm that large (as large as several tens of centimetres in diameter) dome-shaped bubbles can be reproduced for measured rheological properties. We investigate the nucleation and expansion of holes in the hemispherical fluid sheet by photogrammetry in the field and in laboratory experiments with natural mud. Multiple near-simultaneous film ruptures have nucleation and growth dynamics that share some features of first-order phase transitions. The rate of hole expansion is well-described by a generalized version of the Taylor–Culick model [11,12], resulting in a terminal speed which depends only on the fluid density, surface tension and thickness of the fluid film and is independent of the mud rheology.

## 2. Material and methods

### 2.1. Field sites

**Figure 2.**
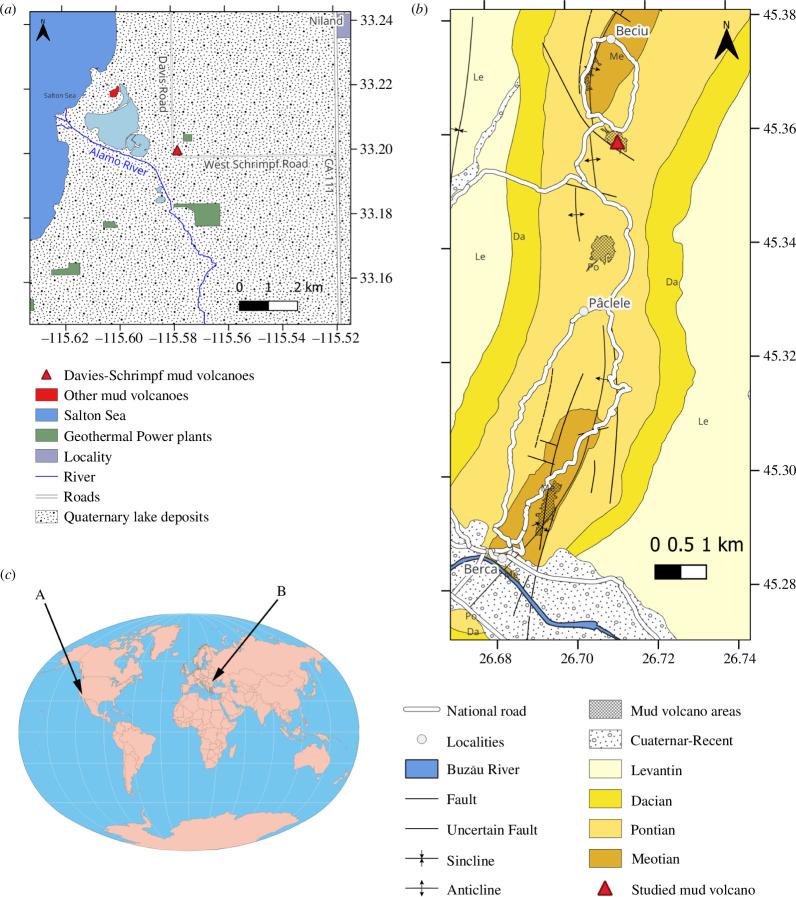
Geological maps of the studied areas. (*a*) Imperial Valley, California, USA, based on USGS [13] data. (*b*) Berca–Beciu–Arbănași mud volcano area (Romania), using data from Ciocârdel [14]. The mapped deposits' age and lithology are Maeotian (from 8.4 to 6 Ma, marl and sandy marl), Pontian (from 6 to 4.7 Ma, marl and sand), Dacian (from 4.7 to 4 Ma, marl and sand), Romanian (from 4 to 2.58 Ma, mostly sand) and Quaternary (from 2.58 Ma to present, sand and gravel). On both maps, the coordinate system is in geographic latitude/longitude. (*c*) Geographic location of both sites.

Our investigations focused on two sites with mud volcanoes: Imperial Valley, California, USA, and Pâclele Mici in the Buzău region, Romania (figure 2). Laboratory experiments were performed using the mud collected at the Imperial Valley, while field measurements were made at Pâclele Mici.

#### 2.1.1. Imperial Valley, California, USA

Mud was collected from the Davis–Schrimpf mud volcanoes near the Salton Sea (figure 2*a*) in the Imperial Valley, California, USA. This region is a sediment-filled tectonic basin associated with the extensional stepover (normal faulting) between the dextral (right lateral, strike-slip) San Andreas and Imperial faults. The eruption of mud and fluids at the ∼ 2–3-m tall gryphons is the surface manifestation of the Salton Sea Geothermal System, where metamorphic decarbonation releases a gas that ascends upwards along fault-controlled pathways [15,16]. Temperatures within the exposed mud pools in the gryphons range from 18°C to 70°C [17–20]. The solids are sourced from lacustrine and deltaic deposits [15] and are dominated by quartz, feldspar and clay minerals (illite, montmorillonite and kaolinite) [17,19,21]. The median grain size of the mud is 24 µm [19]. The gas-driving mud ascent and eruption is primarily CO_2_ with very minor amounts of methane [22]. We collected mud from open vents on the Davis–Schrimpf mud volcanoes and stored it in 20-l sealed buckets prior to being used for laboratory experiments.

#### 2.1.2. Pâclele Mici region, Romania

The Pâclele Mici mud volcano field [14] is located in the southern part of the Eastern Carpathian Mountains (Romania) along the Berca–Berciu–Arbănași anticline (figure 2*b*), a region rich in hydrocarbon resources. The field is in the proximity of the southeastern bending zone of the Carpathians, in the Carpathian Foredeep [23]. Gas and connate water (from Meotian deposits) rise to the surface along the faults and mix with Pontian marl and phreatic water at shallow depths [14].

The depth from which the erupting mud originates is debated. The mud may arise from Middle Miocene deposits of about 3-km depth [24]. However, an argument favouring a shallower depth of mud origin is that mud volcano activity intensifies during or after heavier rains, indicating a shallower water source [25,26]. The solids in the mud are dominated by quartz, kaolinite, illite and calcite, identified using X-ray diffraction (electronic supplementary material, figure S4). The results from ICP-MS analysis of the mud for metals and semimetals are provided in electronic supplementary material, table S3. While letting the mud decant, a supernatant liquid will appear. This liquid has a significant amount of dissolved salts [27] (electronic supplementary material, table S4, with ICP-MS analysis, electronic supplementary material, table S5, for anions and chemical parameters). The supernatant also contains various organic components identified by GC-MS (electronic supplementary materials, §S1.6), which are common in mud volcanoes [28].

Most of the mud volcanoes in Pâclele Mici display continuous gas bubbling and mud overflow, with small rhythmical eruptions. The erupting gas has a methane concentration of over 95% [29] (electronic supplementary material, figure S2). The grain size distribution was measured with a Horiba laser scattering particle size analyser (electronic supplementary material, figure S3) and the characteristic size of the solid particles in the mud is of the order of 10 µm. The high-speed video recordings of bubbles bursting were made in the highest vent of the central crater of the Pâclele Mici region [30]. One of our samples was collected from the periphery and the other from the centre of the ∼ 5-m-diameter central crater. The samples were stored in 0.5-l glass bottles having a glass lid sealed with a rubber ring prior to being used for rheology and surface tension measurements.

Note that distinct craters at the Pâclele Mici site have muds with different rheological properties, as a result of their different water content. We examined only bubble formation, breakup and mud properties in the central crater.

### 2.2. Characterization of mud rheology

We characterized the rheology of mud samples from the Imperial Valley (USA) and from Pâclele Mici (Romania). Mud is a non-Newtonian fluid, with yield stress and shear-thinning behaviour (e.g. [31]). For the purposes of numerical modelling of bubble ascent (described in §2.6), the stress and strain-rate measurements were fit with a Carreau–Yasuda rheological model (e.g. [32]). The effective viscosity of the mud $\left(\mu_{m}\right)$ is given by


(2.1) $$\mu_{m}=\mu_{0}{\left[1+{\left(\lambda \dot{\gamma }\right)}^{2}\right]}^{(n-1)/2}.$$

Here, $\mu_{0}$ represents the zero shear-rate viscosity, and $\dot{\gamma }$ shear rate, n is the power law index and λ is the consistency. The rheological measurements using a Herschel–Bulkley model (power law fluid with yield stress) are shown in electronic supplementary material, figure S1.

#### 2.2.1. Imperial Valley mud

We measured the rheology of three mud samples with different water contents (38.5, 39.5 and 39.7 wt%, electronic supplementary material, table S1) that were used in our experiments, described later. The water content of each mud sample was determined by weighing the sample before and after drying in an oven at 120°C for 3 h.

Rheological properties were measured using an Anton Paar MCR 302 rheometer with a parallel plate (PP25) geometry with a sample gap of 1 mm at 25°C. We first performed a ramped pre-shear (0.1–100 s^−1^) while tracking viscosity. Following this, we measured the shear-stress–shear-rate relationship under both controlled-stress (ramping up from 1 to 200 Pa) and controlled-rate (0.1–1000 s^−1^) conditions.

#### 2.2.2. Pâclele Mici mud

We measured the rheology of two mud samples with different water contents (55.8 and 49.5 wt%), the first one (D2 in electronic supplementary material, table S1) from the centre of the central crater and the second one (D1 in electronic supplementary material, table S1) from its periphery. Note that the bubbles shown in figure 1 form in the central region. The dry matter content/water content was determined by weighing the sample before and after drying in an oven at 105°C until the mass no longer changed.

The rheological properties of Pâclele Mici mud were measured with an Anton Paar Physica MCR301 rheometer under a parallel plate (PP25/S) geometry with a sample gap of 0.5 mm. The samples were pre-sheared by applying a steady rotation (shear rate 100 s^−1^ for 30 s). Subsequently, the shear stress was recorded as a function of shear rate by stepwise increasing the shear rate from 0.01 to 100 s^−1^, followed by a stepwise decrease to 0.01 s^−1^. At each step, the mud sample was first sheared for 10 s. The measurements were performed three times, and each measurement was repeated twice at 15°C and 25°C.

### 2.3. Characterization of mud surface tension

Surface tension was measured for the Pâclele Mici samples. The mud was too viscous to measure its surface tension using the pendant drop or Wilhelmy plate method. Instead, the surface tension measurements were performed by using model materials which were prepared by diluting the natural (original) mud by its dispersion (liquid) phase.

Approximately 500 ml of the original mud was centrifuged with a Hermle Z 36 HK centrifuge (5000 rpm, 20 min). Subsequently, we systematically measured surface tension as a function of water content, starting with the pure supernatant. Surface tension was measured at 25°C with a KRÜSS DSA30 device using the pendant drop method. We measured the surface tension of the pure supernatant and sequentially added the original mud to it by mixing until the surface tension measurement was no longer possible. The surface tension values (72 ± 1 mN/m) were stable and close to the surface tension of water until the water content of the mixture decreased to approximately 82 wt% (electronic supplementary material, table S2). A decrease in the surface tension values was observed when the water content decreased to 78 and 74 wt% (67 ± 1 and 56 ± 4 mN/m). Below this water content, the measurements could not be performed because the fluctuations and uncertainties became excessively large.

From the measurements (electronic supplementary material, table S2), the resultant values for the most concentrated samples (with 78 and 67 wt% water content) were averaged and used in numerical simulations of bubble ascent, described in §2.6. We assumed that this value is representative of the original undiluted mud, and we assume that the surface tension of Imperial Valley mud has a similar value as well. The value of surface tension did not have a noticeable influence on the simulation results because the bubbles rise in the large Eötvös-Bond number (ratio of buoyancy to surface tension forces) limit.

### 2.4. Laboratory experiments with Imperial Valley mud

We carried out laboratory experiments to study the expansion and rupture of bubbles in Imperial Valley mud (figure 1*c*). The goal of these experiments was not to specifically replicate bursting bubbles observed in the field, in part because there are too many uncertainties in properties at field conditions. Rather our goal is to probe the effect of rheology on the dynamics of bubble ascent and rupture to better understand these processes. However, the dynamics seen in the lab at least qualitatively resemble those in the field. We sequentially diluted the mud with water in a set of eight experiments (identified as E1, E2, ..., E8). Aliquots of mud from each of the eight experiments were extracted for the rheology and water content measurements. The rheology of mud is strongly dependent on the water content, and we carried out experiments with water contents of 38.5−39.7 wt%, corresponding to a factor of ∼2 variation in consistency (electronic supplementary material, table S1).

Air was injected into the base of a 14.6-cm inner diameter circular cylinder filled with mud to a depth of 28 cm. The dynamics of bubble expansion and bursting were imaged using a Phantom v1210 high-speed camera recording at 10 000 frames per second (fps). The resolution of the images is approximately 0.17 mm/pixel, determined by using the inner diameter of the cylinder in the images. The camera was positioned to look vertically downward upon bursting bubbles. The size and shape of holes within the expanding film were measured using marker-controlled watershed segmentation in the MATLAB Image Processing Toolbox. We extracted the dimensions of best-fitting ellipsoids to each hole for each video frame, recognizing that the size and shapes of holes away from the apex of the bursting bubble may be subject to some uncertainty owing to optical distortion owing to the viewing angle. We manually counted the number of holes from the video recording. This analysis and other measurements were made after all the experiments were performed and the combination of hole measurements and counting, and rheological and water content measurements, could only be successfully performed for experiments E1, E2 and E6 with water contents of 38.5, 39.5 and 39.7 wt%, respectively.

### 2.5. Field observations at Pâclele Mici

Images of bubble evolution were collected using a Sony Cybershot DSC-RX10 III high-speed camera at 1000 fps. A measuring tape was placed in the vicinity of the emerging bubbles for scale. To measure the retraction speed of holes, we used the openCV module in Python to detect ellipses in sequences of video frames, using the maximum radii of the detected ellipses as a proxy for the hole radii. We converted the number of pixels to centimetres based on the measuring tape that is present in each video recording. The image resolution is approximately 0.18 mm/pixel. Such measurements contain some level of uncertainty, which stems from several reasons: the nearly circular holes appear to be distorted owing to the viewing angle, the distance between the camera and the hole is not identical owing to the shape of the bubble, and the image depth and perspective cannot be accounted for owing to the lack of additional visual cues. Twelve holes were chosen to be traced from three different video sequences. Two basic criteria were used to select them. First, we require that there were no holes in close proximity to each other and that holes retracted long before the mud film began to disintegrate. The tracing of ellipses was terminated once the hole expansion started to interfere with neighbouring holes, or when lighting and shadows in the images prevented the software from accurately detecting an elliptical shape in an automated manner.

### 2.6. Numerical models of bubble ascent

Numerical simulations were conducted employing the parameter set specific to the mud in the Pâclele Mici mud volcano. These parameters (viscosity, flow index and consistency of mud), detailed in electronic supplementary material, table S6, were determined from measurements of mud rheology (§2.2).

Because mud is opaque, the geometry and ascent of bubbles prior to rupture cannot be visualized directly. Numerical models were thus performed to study the dynamics of a gas bubble rising inside a mud pool. We solve the incompressible Navier–Stokes equations for both the gas and mud phases and treat the presence of the interface between the two phases using a volume-of-fluid (VOF) approach. The mud viscosity is parameterized using the Carreau–Yasuda rheological model [32]. Although mud is a non-Newtonian fluid with a yield stress, there are significant challenges associated with capturing yielding in numerical models. In the case of yield stress fluid, the substance behaves like a solid up to a specific stress, above which it can deform and flow as a fluid. The viscosity is effectively infinite for stresses below the yield stress. We have adopted a rheological model that is suitable for use in numerical models while retaining some similarity to rheological models that do include yield stress. In the Carreau–Yasuda model used here, the yield stress fluid’s effectively infinite viscosity at zero strain rate is replaced with a finite (but large) value ( $\mu_{0}$ in equation 2.1). A complete description of the governing equations, numerical approach and material properties is given in electronic supplementary material, §S3. The material properties were chosen based on sample D2 from the central vent at Pâclele Mici, which is the same location as the bubbles shown in figure 1*b*.

## 3. Results

### 3.1. Mud rheology

**Figure 3.**
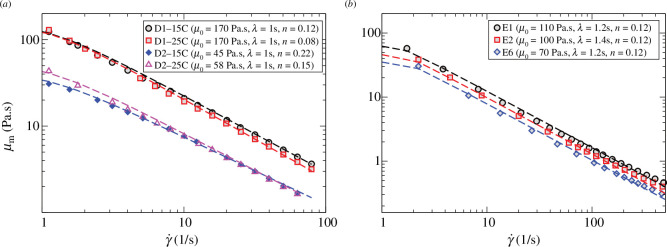
Rheological measurements for Pâclele Mici mud (*a*) and Salton Sea mud (*b*). The curves show the Carreau–Yasuda rheological model [32], with parameters given in the legends.

We fit the rheological measurements with the Carreau–Yasuda rheological model, a generalized power law model that we used in the numerical simulations of bubble ascent. The Carreau–Yasuda fits to the data are shown in figure 3. For completeness, we also show fits of a Herschel–Bulkley model with yield stress to the data (electronic supplementary material, §S1.1).

### 3.2. Numerical simulations of ascending gas bubbles

**Figure 4.**
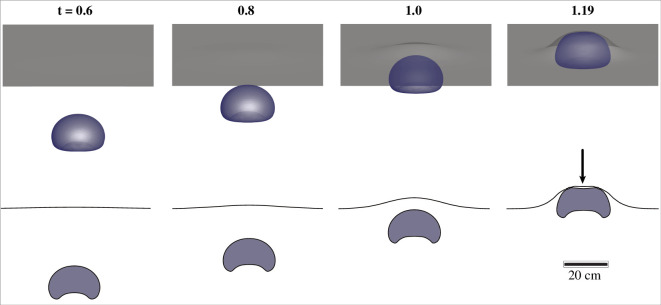
Results from the numerical simulations. Temporal evolution of bubble shape as it approaches the free surface, modelled using the Carreau–Yasuda rheological model [32]. The top and bottom rows depict three-dimensional and cross-sectional views, respectively. In the cross-sectional view, the black curve indicates the free surface and the bubble is shown in blue. The number at the top of each panel is the numerical time in seconds with t=0 the instant when the bubble starts to rise with a hemispherical morphology from a location 7R beneath the undisturbed free surface. Here, R denotes the equivalent spherical radius of the bubble (= 20 cm). The rest of the parameters are given in electronic supplementary material, table S6.

The numerical simulations of gas bubble ascent show that gas bubbles take the shape of a dimpled ellipsoidal cap (figure 4). This is consistent with the behaviour seen in Newtonian fluids at similar values of the Reynolds and Eötvös-Bond numbers [33]. However, owing to the non-Newtonian rheology of the mud, the bubble does not attain a steady shape, and the dimpled region continues to expand. The interface curvature is largest near the edge of the dimple on the aft side of the bubble. The curved interface on the bottom of the bubble is also visible in the field observations as the bubble bursts in figure 1*c*, confirming the bubble shape obtained in the simulations. In the numerical models, there is a local thickening of the film at the top of the ascending bubble (indicated with an arrow in figure 4), similar to the dimples that occur during film drainage between coalescing drops and the impingement of drops on surfaces. Although the dimple at the apex of the bubble cannot be measured in experiments or field observations, there is indirect evidence for its existence. In both the field and laboratory, when bubbles rupture, the first holes are offset from the apex of the bubble, which suggests that the fluid film is not necessarily thinnest at the apex. This stands in contrast to the dynamics of an expanding bubble in a Newtonian fluid, where the film is thinnest at the apex of the bubble [34]. Film rupture is not captured in the models owing to the vast difference in the spatial scales of bubble size relative to the film thickness at the point of rupture, which we are not able to resolve numerically.

### 3.3. Formation of holes

In both field observations and laboratory experiments, at a certain stage of bubble growth, multiple holes form or ‘nucleate’. The first holes do not form at the apex of the bubble, as in similar experiments with Newtonian fluids, but slightly offset (figure 1*c*), likely owing to the thickening of the fluid film at the apex, predicted by the numerical models and arises from the non-Newtonian behaviour of the mud (figure 4).

**Figure 5.**
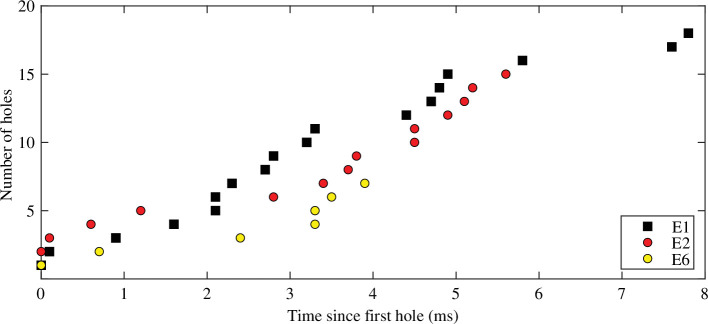
Number of holes versus time for laboratory experiments. The colours correspond to laboratory experiments carried out with different water contents. Water content increases from E1 (38.5%) to E2 (39.5%) to E6 (39.7%).

Once the first hole forms in the thinning fluid sheet, others form in quick succession (multiple holes appear within less than 1 ms). In laboratory experiments performed using the Imperial Valley mud with different water content, where a camera was positioned directly above the bubble (refer figure 1*c*), the number of holes appears to grow linearly in time (figure 5). The less-viscous muds formed fewer holes and holes nucleated more slowly.

### 3.4. Expansion of holes

**Figure 6.**
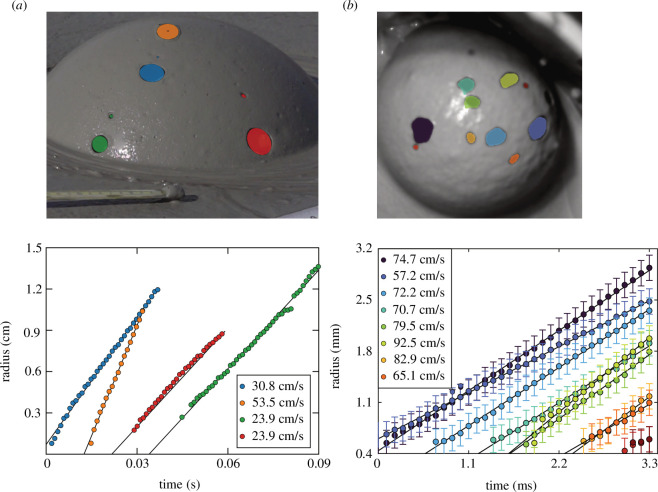
Speed of hole expansion extracted from video frames for field videos at Pâclele Mici (*a*) and laboratory measurements from experiment E1 with Imperial Valley mud (*b*). (*a*) Top: ellipsoidal fits of approximate starting and final positions of the holes. Bottom: measurements of the maximum radius as a function of time alongside a fit. (*b*) Top: coloured regions indicate nucleated holes. Bottom: hole radius as a function of time. The legends in both panels indicate the terminal speed extracted from the data; the shaded regions in the images are coloured in accordance with the corresponding colours used for the radii on the bottom part of each panel. In the bottom left panel, the uncertainty in radius (1 pixel) is smaller than the symbols used. The error bars in the lower right panel indicate an uncertainty of 1 pixel. Note that the image in the top panel in (*a*) is edited digitally to show the holes as they are superimposed on the bubble at t=0 ; in subsequent times, sagging of the bubble structure and multiple additional holes develop. The expansion speed converges to a constant value that differs within the same bubbles and between bubbles.

After their appearance, the holes quickly expand. In figure 6, we show the evolution of hole size for selected natural bubbles (A) and for bubbles in laboratory experiments (B). The expansion rates of holes in laboratory experiments and field observations appear to reach a constant speed of 24−93 cm/s after a few milliseconds that we refer to as the long-time limit. We postulate that this approach towards a constant speed is in agreement with the classical Taylor–Culick theory [11,12], according to which the constant speed can be derived through a series of arguments based on the conservation of momentum within the rim of the sheet [11,12]. The generalization of the Taylor–Culick theory presented in §4.3 directly looks at the momentum equation for the whole sheet but we show how one can arrive at the same result independently of the form of the stress tensor for the fluid.

In our field observations, the speed of the hole expansion decelerates in the first milliseconds after the hole formation but later approaches a constant asymptotic value. This can be seen most clearly for the blue curve in figure 6*a*. This type of behaviour can be attributed, at least partly, to the initial shape of the rim of the hole when it is created [35], the rheological properties of the fluid, or possibly owing to the bubble having locally thinner walls. Our laboratory experiments also hint to a more rapid hole expansion during the initial stages (see figure 6*b*), but typically this effect is too brief to be investigated precisely, especially given that the photogrammetry is most uncertain when hole radii are small.

The rate of steady hole expansion differs considerably among holes growing on any given bubble (figure 6) and between bubbles in Imperial Valley versus Pâclele Mici mud. Long-time hole expansion speeds in the Imperial Valley mud are more rapid (57–93 cm/s) than those observed in the Pâclele Mici mud (24–54 cm/s).

Owing to inertia, the bubbles are still expanding during the initial phase of hole growth. However, we do not find evidence that the ongoing expansion of the bubble has an observable effect on the speed of hole expansion.

## 4. Discussion

### 4.1. Bubble shape

The shapes and dynamics of gas bubbles have attracted the attention of scientists since the early 20th century owing to their natural beauty and importance to engineered systems. The formation of large (tens of cm) bubbles at the surfaces of mud volcanoes (figure 1) implies that large gas bubbles can ascend through viscous mud without breaking up into smaller bubbles.

A regime map has been developed to understand and categorize the diversity of bubble shapes, including spherical, oblate, wobbling and skirted bubbles for Newtonian fluids (e.g. [33]). This regime map has been further expanded through extensive numerical simulations and experiments, incorporating breakup bubbles into the map [36,37]. There is no generalized equivalent regime map for non-Newtonian fluids, though a number of studies have examined controls on bubble shape for specific classes of non-Newtonian fluids. For example, Handzy & Belmonte [38] explored the dynamics of rising bubbles within an aqueous solution of cetylpyridinium chloride and sodium salicylate (Maxwell-type viscoelastic fluids). By varying the concentrations and temperature, they identified various regimes, including those without a cusp, steady cusp and oscillatory bubbles. Xu *et al*. [39] devised a regime map illustrating different dynamics of an air bubble rising in PAAm and xanthan gum in the Reynolds number and Eötvös-Bond number space. By varying the air bubble volume, Polyres & Vidal [40] observed a small spherical bubble, a bubble exhibiting a cusp at its rear, bubble deflection and bubble fragmentation with a fingering instability. Although the fluids investigated in our study are non-Newtonian, the Newtonian regime map helps us to understand the qualitative behaviour of bubbles rising in non-Newtonian fluids. It is noted that the Carreau–Yasuda model does not replicate the cusp shape observed in the previous studies. Extensive numerical simulations that account for viscoelasticity are necessary to identify all the intriguing characteristics of mud bubbles in this context.

The bubble dynamics can be described using four dimensionless groups. The Galilei number ( $Ga=\rho_{m}g^{1/2}R^{3/2}/\mu_{m}$ ) measures the relative importance of buoyancy and viscous forces. The Eötvös-Bond number ( $Bo=\rho_{m}gR^{2}/\sigma$ ) is the ratio of buoyancy and surface tension forces. The density ratio ( $\rho_{g}/\rho_{m}$ ) and the viscosity ratio ( $\mu_{g}/\mu_{m}$ ) express the contrast in physical properties between the bubble and ambient fluid. Here, $R=(3V/4\pi {)}^{1/3}$ represents a characteristic radius of the bubble with V the bubble volume, σ is the interfacial tension and $\left(\rho_{g},\mu_{g}\right)$ and $\left(\rho_{m},\mu_{m}\right)$ denote the dynamic viscosity and density of the gas and continuous phases, respectively.

The bubbles studied here have low ( O(1) ) Ga and high ( $O(10^{3})$ ) Bo , thus exhibiting symmetrical oblate or dimpled shapes. At such a high Eötvös-Bond number ( Bo ), the influence of surface tension is negligible when compared with the force of gravity. Thus, despite their larger size, gas bubbles in mud do not disintegrate and take on an oblate or dimpled shape, as depicted in figure 4. Because the viscosity of water is $∼4\times 10^{4}$ times less than that of mud, the Galilei number of bubbles of similar size in water is orders of magnitude larger, leading to bubble breakup [36].

In the numerical simulations of bubble ascent, as the bubble impinges upon the free surface, a dimple develops at the uppermost part of the bubble and a thickened region develops in the fluid sheet above the dimple. Similar features are observed during the impingement of bubbles on a rigid interface and during bubble coalescence [41], during the ascent and bursting of bubbles in a Hele–Shaw cell with shear-thinning fluids [40], and in numerical models of bubble ascent and interaction in Bingham fluids [42]. This feature cannot be directly observed in our experiments because we cannot measure film thickness. Although we do not directly observe a dimple at the top of the bubble or the closely related thickening of the film in the experiments and field observations, we note that holes do not form preferentially at the apex of bursting bubbles, suggesting that the apex is not the thinnest part of the fluid sheet. The development of this dimple appears to be related to rheology, perhaps associated with the localization of strain rate away from the stagnation point directly above the bubble. In simulations with Newtonian rheology (shown in electronic supplementary material, figure S10), we do not observe any dimpling at the top of the bubble and the film is thinnest at the apex.

### 4.2. Hole formation

When a bubble impinges upon the free surface, the fluid overlying the bubble is deformed upward into a hemispherical thin sheet that undergoes thinning owing to the expansion of the gas within the bubble and the drainage of the fluid film. When the film becomes sufficiently thin, holes start to form at a constant rate (figure 5). As holes expand and coalesce, the fluid film undergoes a reorganization into filaments which then break into droplets (figure 1). Similar phenomenology has been observed in the fragmentation of rapidly expanding liquid shells [43], lava bubbles (figure 1) and thinning viscous sheets [44]. In the flame-driven shell expansion experiments of [43], the fluid rheology is Newtonian, suggesting that the complex rheology of mud and magma is not a prerequisite for bubble disintegration through the rapid formation of multiple holes.

Several mechanisms have been proposed to nucleate holes, including a Rayleigh–Taylor instability within an accelerated fluid interface [43], the Marangoni instability [45] and the capillary (Plateau–Rayleigh) instability [46,47]. Of these mechanisms, the Rayleigh–Taylor instability within an accelerating fluid film appears broadly consistent with the phenomenology of bubble bursting in mud and magma. Instabilities in the fluid film are capable of explaining the formation of multiple holes even in a homogeneous fluid. However, we cannot exclude the possibility that hole formation is affected by the concentration of stresses around larger solid particles or by the deformation history of the mud.

Hole formation and evolution in fluid sheets shares some common features with the nucleation process in first-order phase transitions. For example, holes must achieve a critical radius to start growing and not disappear [4,45,48–50] but their value is found to be smaller than the thickness of the film [51,52]. When a phase transition occurs, certain properties of the physical system, characterized by an order parameter, are suddenly and qualitatively altered as an external factor, and the control parameter is smoothly varied. In first-order phase transitions, the new phase may appear through nucleation, a random process where a critical extent of the new (more stable) phase has to form in order not disappear but to start growing at the expense of the old (less stable) phase. In heterogeneous nucleation, the presence of impurities can lower the free energy barrier required for the formation of the new phase having this critical extent.

The sequence of events during the rupture of mud bubbles shows some similarities with a phase transition through heterogeneous nucleation. Once the bubble reaches the surface, the film of mud surrounding the bubble thins through drainage and stretching. As the thickness of the film considered the control parameter of the system, decreases to some critical value, the fluid film becomes unstable and holes will emerge. Achieving the critical nucleus size may be influenced by the inhomogeneities of the mud. Note that the later stage of bubble bursting resembles a phase separation: the mud sheet separates into zero-density holes and thickening filaments.

This scenario can result in two outcomes depending on whether nucleation or hole expansion dominates. If the alteration of the control parameter (the film thickness) quickly drives the system far into the unstable region, several holes will nucleate in a short time frame provided that hole expansion is sufficiently slow that it does not destroy the fluid film in this time frame. This is the case observed with mud bubbles [53]. In contrast, when hole expansion dominates over nucleation, we hypothesize that only one hole will form.

### 4.3. Hole expansion rate

The stability and growth of holes in fluid films has been studied since the nineteenth century, when it was observed that hole expansion approaches a constant rate, which was found independently by Taylor [11] and Culick [12]. Taylor and Culick corrected an earlier analysis by Dupré [54] who obtained an expansion rate from energy arguments, but neglected viscous dissipation. In accordance with the Taylor–Culick theory, the terminal speed of the hole expansion depends only on the film’s density, thickness and surface tension. Subsequently, it has been discovered that the initial phase of hole growth may not be linear during the early stages of retraction, but is exponentially accelerating [1,55]. This effect was documented in very viscous fluids, when the Ohnesorge number (a dimensionless ratio of viscous forces to inertial and surface tension forces), defined as $Oh=\mu_{m}/\sqrt{\rho_{m}\sigma H}\gg 1$ , so that the hole expansion is considerably slowed down (*H* is the thickness of the sheet).

For the present study, Oh is of order unity (see the following text), favouring a rapid transition towards the terminal speed [35], which is also confirmed by the data shown in figure 6. In accordance with estimates from experimental measurements, taking σ=0.07 N/m, ρ=1400 kg/m^3^ and $u_{c}=0.6$ m/s, the terminal speed of the Taylor–Culick theory, we deduce from equation (4.13) that $H\approx 3\times 10^{-4}$ m. The characteristic shear rate is thus $\dot{\gamma }=u_{c}/H\approx 2000$ s^−1^ for which the effective viscosity is about 0.2 Pa s (using data from §3.1). Using the definition of Oh and equation (4.13), we deduce $Oh=\mu u_{c}/(\sqrt{2}\sigma )$ so that we finally get Oh≈1 . The relationship describing the exponential phase includes a characteristic time, which is proportional to the dynamic viscosity and thickness, while inversely proportional to the surface tension of the fluid [35]. The expansion speed asymptotically approaches a constant value regardless of the viscosity of the fluid.

**Figure 7.**
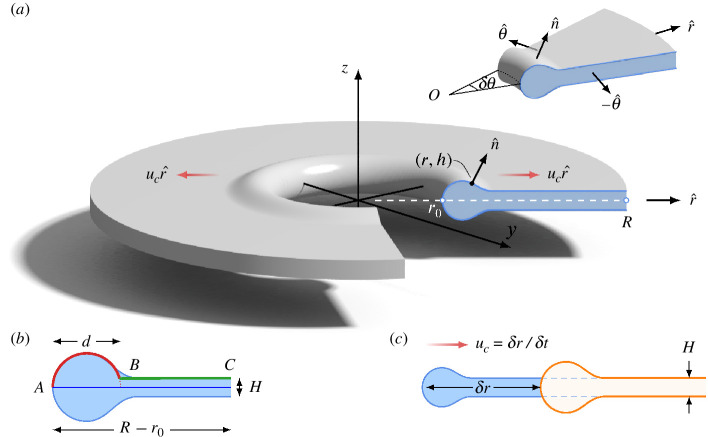
(*a*) Rendering of an axisymmetric sheet of thickness H retracting with speed $u_{c}$ , showing its cross section (blue shaded region), which is described by the profile z=h(r,t) above the axis of symmetry for $r_{0}\leq r\leq R$ ; $\hat{n}$ is the outward unit normal to the free surface of the sheet. Inset: schematic illustration of the volume over which the integration is carried out. (*b*) Partition of the cross-section for estimating the length of the profile ABC: AB is a circular arc of approximate length (πd−H)/2 ; BC is a straight segment of approximate length $R-r_{0}-d$ (*c*) Within time δt , the tip of the rim moves by a distance $\delta r=u_{c}\delta t$ ; the mass of the fluid accumulated in the rim during that time is $\delta m\approx 2\pi r_{0}\rho H\;\delta r$ .


The measurements of hole expansion speed (figure 6) show that for both laboratory and field measurements, the expansion rate appears to quickly reach a steady value. Here, we will show that a constant retraction speed is reached in the limit when the hole has expanded sufficiently, a speed that is independent of the fluid rheology. To show this, we assume that we have a large axisymmetric liquid sheet that retracts under the influence of capillary forces acting at the edge of its rim, illustrated schematically in figure 7*a*. Gravitational effects are neglected because we are interested in the intermediate stages of hole formation; at later stages, the thin film structure collapses and we can no longer exploit any symmetries in the system. This means that gravity manifests itself at much longer times compared with the time to reach the terminal speed. Under these assumptions, the flow is governed by the Cauchy momentum equation, namely

(4.1) $$\rho_{m}\;\frac{Du}{Dt}=\nabla \cdot T,$$

where u is the fluid’s velocity, T is the (Cauchy) stress tensor of the fluid, and Du/Dt is the material time derivative of u . The stress tensor is also typically expressed as T=−pI+τ , where p is the pressure, I is the identity tensor so that the pressure acts isotropically within the fluid and τ is the deviatoric stress tensor that captures viscous (including potentially non-Newtonian) effects.

Exploiting the assumption that the flow is axisymmetric, we may carry out an integral over a representative volume V(t) , so that


(4.2) $$\rho_{m}{∭}_{V(t)}r\frac{Du}{Dt}\;dz\;dr\;d\theta ={∭}_{V(t)}r\;\nabla \cdot T\;dz\;dr\;d\theta ,$$

as expressed in the usual cylindrical coordinate system (r,θ,z) . This volume represents a narrow slice of the sheet, with radial extent $r_{0}(t)\leq r\leq R$ , spanning a small azimuthal angle δθ so that $-\delta \theta /2+\theta_{0}\leq \theta \leq \theta_{0}+\delta \theta /2$ and $\theta_{0}$ arbitrary (see figure 7*a*). Here, $r_{0}(t)$ is the radial tip position of the sheet and R is some generally large (or even infinite) distance, where the sheet terminates, so that it is not impacted by the dynamics in the vicinity of the hole. On the left-hand side of equation (4.2) , we apply the Reynolds transport theorem which allows us to move the time derivative outside the integral sign, whereas on the right-hand side, we apply the Gauss–Ostrogradskij divergence theorem to reduce it to a surface integral over the free surface of the slice, denoted by S(t) , so that


(4.3) $$\frac{dP}{dt}=\iint_{S(t)}T\cdot \hat{n}\;ds,$$

where the net (radial) contributions coming from the surface integrals on either side of the narrow slice are negligible owing to the smallness of δθ . Here, $P=\rho_{m}{∭}_{V}ru\;dz\;dr\;d\theta \approx \rho_{m}\delta \theta \iint_{S}ru\;dz\;dr$ denotes the total momentum vector within the narrow slice of the sheet and $\hat{n}$ is the outward unit normal to the free surface of the film, S(t) , see figure 7*a*. Similar principles have been employed by Bohr and Scheichl [56] for the transport of energy in a moving fluid. On the free surface, S(t) , surface tension forces balance viscous stresses, so that the normal stress boundary condition reads $T\cdot \hat{n}=-\sigma \kappa \hat{n}$ , where σ is the surface tension and κ is the curvature of the boundary S(t) so that equation (4.3) becomes


(4.4) $$\frac{\;d}{\;dt}P\left(t\right)=-\iint_{S\left(t\right)}\sigma \kappa \hat{n}\;ds.$$

We see that viscous terms do not appear explicitly in equation (4.4), since the source of momentum is the unbalanced surface tension force at the rim of the retracting sheet, as argued in related works [11,12,35,57]. However, we emphasize that viscosity is not neglected, and viscous dissipation does occur within the retracting sheet [12,57]. By exploiting the top-bottom symmetry of the sheet (figure 7*a*), we may describe its axisymmetric profile above the xy -plane by z=h(r,t) so that $ds=\delta \theta r\sqrt{1+h_{r}^{2}}\;dr$ , and


(4.5) $$\kappa =\nabla \cdot \hat{n}=-\left[\frac{h_{rr}}{{\left(1+h_{r}^{2}\right)}^{3/2}}+\frac{h_{r}}{r{\left(1+h_{r}^{2}\right)}^{1/2}}\right]\;\text{and}\;\hat{n}=\frac{-h_{r}\hat{r}+\hat{k}}{\sqrt{1+h_{r}^{2}}},$$

where $h_{r}=\partial h/\partial r$ and $h_{rr}=\partial^{2}h/\partial r^{2}$ . Hence, equation (4.4) simplifies


(4.6) $$\frac{\;d}{\;dt}P\left(t\right)=-2\delta \theta \;\sigma \hat{r}\int_{r_{0}}^{R}\left[\frac{rh_{r}h_{rr}}{{\left(1+h_{r}^{2}\right)}^{3/2}}+\frac{h_{r}^{2}}{{\left(1+h_{r}^{2}\right)}^{1/2}}\right]dr,$$

where, at leading-order in δθ , $\hat{r}$ may be taken outside the integral sign. In equation (4.6), the factor of two accounts for both the top and bottom parts of the sheet, where, owing to symmetry, the $\hat{k}$ ( z -directed) component of 𝑷(t) vanishes. By applying integration by parts, equation (4.6) simplifies to


(4.7) $$\begin{array}{ll} \frac{d}{dt}\;P\;(t) & =2\delta \theta \;\sigma \hat{r}\int_{r_{0}}^{R}\left[r\frac{\partial }{\partial r}\left(\frac{1}{(1+h_{r}^{2}{)}^{1/2}}\right)-\frac{h_{r}^{2}}{(1+h_{r}^{2}{)}^{1/2}}dr\right]\\ & =2\delta \theta \;\sigma \hat{r}\left[R-\int_{ro}^{R}\sqrt{1+h_{r}^{2}}\;\;dr\right] \end{array}$$

noting that, in deducing equation (4.7), we assumed that $h_{r}\to \infty$ as $x\to r_{0}$ and $h_{r}\to 0$ as r→R . The arguments leading to equation (4.7) justify rigorously why viscous effects do not influence the terminal velocity, unlike in the aforementioned works of Taylor and Culick, whose arguments relied on their sharp physical intuition about the system [11,12].

The integral term in equation (4.7) corresponds to the length of the profile of the sheet, which may be estimated by adding the length of the (assumed) circular arc for the profile near the rim and the flattened part of the sheet, namely


(4.8) $$\int_{r_{0}}^{R}\sqrt{1+h_{r}^{2}}\;dr\approx \left(\frac{\pi }{2}-1\right)d(t)-\frac{H}{2}+R-r_{0}(t),$$

where H is the thickness of the sheet and d(t) is the (growing) diameter of the rim, see figure 7*b*. Hence, equation (4.7) may be estimated as follows


(4.9) $$\frac{\;d}{\;dt}P\left(t\right)\approx 2\delta \theta \;r_{0}(t)\sigma \hat{r}\left[1+\frac{H}{2r_{0}(t)}-\left(\frac{\pi }{2}-1\right)\frac{d(t)}{r_{0}(t)}\right].$$

For $r_{0}\gg H$ and $r_{0}\gg d$ , the last two terms in equation (4.9) may be neglected so that as the hole grows sufficiently large


(4.10) $$\frac{\;d}{\;dt}P\left(t\right)\to 2\delta \theta \;r_{0}(t)\sigma \hat{r}.$$

In experiments, we observe that $r_{0}\gg H$ within milliseconds after the nucleation of the hole. While it is true that the rim diameter d grows with the hole radius $r_{0}$ , a simple scaling argument reveals that $d∼\sqrt{r_{0}H}$ , so that $r_{0}\gg d$ also applies after some initial transients. Hence, we conclude that the retraction dynamics may be adequately described by assuming a planar two-dimensional sheet within a short time of the nucleation of the hole in the low-viscosity (low- Oh ) limit considered here. Thus, independently of the rheology of the fluid, and hence independently of the form of the stress tensor T , the evolution of the sheet’s momentum is governed by equation (4.7) with the total momentum directed in the r direction. As the hole expands, the rate of change of momentum is governed by equation (4.10), which is equivalent to the planar limit, in the sense that the rate of change of total momentum per unit length approaches 2σ , refer e.g. the work of Savva & Bush [35] on two-dimensional Newtonian viscous sheets. It should be emphasized that the development of this theory does not apply to the limit of high-yield stress fluids, owing to the discontinuous manner in which the fluid behaves. For example, for viscoplastic liquid sheets with a sufficiently large Oh , the plastic nature of the fluid can prevent the retraction of the sheet for high-yield stresses [58]. The yield stress of the muds considered here is clearly low enough that hole expansion is unimpeded.

The rest of the derivation of $u_{c}$ follows the arguments of [11] and [12], by assuming that we have a rim of mass m(t) that collects fluid in an otherwise quiescent film so that


(4.11) $$\frac{\;d}{\;dt}P\left(t\right)=u_{c}\frac{\;dm}{\;dt}\hat{r}=2\sigma \delta \theta \;r_{0}\hat{r}.$$

The mass accumulated in the rim during time δt , δm , is given by $\delta m=\delta \theta \;\rho_{m}r_{0}H\delta r$ , where $\delta r=u_{c}\delta t$ is the distance travelled by the tip of the rim during that time. Hence, the rate of change of the rim mass satisfies


(4.12) $$\frac{\;dm}{\;dt}\approx \frac{\delta m}{\delta t}=\delta \theta \;r_{0}\rho_{m}Hu_{c}.$$

By substituting equation (4.12) in equation (4.11), we may solve for $u_{c}$ so that


(4.13) $$u_{c}=\sqrt{\frac{2\sigma }{\rho_{m}H}}.$$

Hence, consistent with [11,12], the terminal hole expansion rate depends only on the surface tension and the density of the film and is independent of the rheology of the mud. For the measured values of $\rho_{m}$ and σ , we used equation (4.13) to predict the speed of hole expansion as a function of the (unknown) film thickness H (electronic supplementary material, figure S11). We find $u_{c}=0.29$ m/s, comparable to the measured speeds, for H=1 mm.

The constant speed of retraction is a consequence of the balance of inertial and surface tension forces. It is important to note, however, that just as in Newtonian fluids, equation (4.11) accounts for viscous dissipation, but in a manner that is not related explicitly to viscosity [57]. Viscous effects merely affect how the momentum is distributed through the sheet and the time required to reach the terminal speed, $u_{c}$ , but not the value of $u_{c}$ itself [35]. In other words, viscous effects dictate the extent of the region over which energy dissipation occurs. At higher viscosity, the liquid is in motion further away from the retracting tip and viscous dissipation is observed throughout. Hence, no visible rim forms as in the experiments by Debrégeas *et al*. [55], and, as a result, a longer time is required to reach $u_{c}$ . For lower viscosities, the liquid is collected within a retracting rim, which assumes its terminal speed within a short time [12,35]. In such cases, viscous dissipation primarily occurs in the neck region, where a rim of nearly constant speed meets the quiescent part of the sheet [59]. In fact, it has been shown that in the long-time limit, exactly one-half of the surface energy that is introduced into the system owing to the destruction of the sheet is converted into kinetic energy, and the other half is dissipated [12,57,59]. Recently, departures from the classical hole expansion rate (equation (4.13)) have also been observed. For example, Deka and Pearson reported a slow-down of the expansion rate in numerical simulations with finite sheets in the large Oh limit [60], whereas Sanjay *et al*. [59] found that if the ambient fluid is sufficiently viscous, dissipation in the ambient dominates and the hole expansion occurs at a slower constant rate that depends on the viscosity of the ambient fluid [59]. Numerical simulations with viscoplastic sheets, but with a sufficiently low yield stress that retraction occurs, also anticipated the eventual transition to $u_{c}$ , which was nevertheless not fully observed in practice owing to the small size of the computational domain considered [58].

## 5. Conclusions

We investigated the ascent and rupture of gas bubbles in natural mud volcanoes and under laboratory conditions using a combination of field observations, laboratory experiments, numerical simulations, and theoretical models. The phenomenology of bubble bursting in mud is similar to that seen in some lava bubbles (figure 1), highlighting the potential of mud volcanoes to serve as analogues to their more hazardous volcanic counterparts. Numerical simulations of bubble ascent indicate that large (decimetre-sized) gas bubbles ascend as ellipsoidal caps (with a semi-crescent cross-section), and are not expected to break up into cascades of smaller bubbles owing to the high Eötvös-Bond and low Galilei numbers.

The numerical simulations reveal a dimple at the apex of the bubble and a thickening of the fluid film above the dimple, a consequence of the non-Newtonian rheology of the fluid. This numerical finding is supported indirectly. The first holes are assumed to appear at the thinnest part of the fluid film, and indeed, our experiments and observations showed that the sites of the earliest nucleation formed in a ring-like zone around the apex (figure 1).

The pattern formation in the fluid sheet starts with the rapid formation of multiple holes. Hole nucleation occurs within the span of a few milliseconds and has a constant rate. At the same time, the holes expand. The speed of hole expansion approaches a constant value that differs among holes even on the same bubble, likely owing to variations in the thickness of the fluid sheet. The fact that multiple holes can temporally coexist is explained by the relatively fast nucleation and slow growth rates. We pointed out the similarities of this sequence of events to first-order phase transitions.

By considering the total momentum of an axisymmetric sheet as it moves in gas so that the dynamics of the ambient fluid may be neglected, our theoretical model for the hole expansion rate generalizes the results of Taylor and Culick for fluid sheets of arbitrary rheology, including the case of bubbles in aqueous solutions if the bubble is large enough and surface tension gradients are not dynamically significant. We show that the Taylor–Culick theory applies, and thus that the terminal speed of the expansion rate of the hole depends only on the thickness of the fluid sheet, its mass density and surface tension. This rate was found to be independent of fluid viscosity, owing to the internal nature of the associated viscous stresses [35,59]. That viscosity does not influence the speed of retraction can intuitively be explained as follows. Energy dissipation by viscosity is related to strain rate. In the investigated phenomenon, the expansion of the holes does not influence the distant parts of the fluid film, which, over the time scale of the fast hole expansion, can be considered to be steady. As highlighted in related studies, the dissipation of energy occurs in the vicinity of the neck region where the rim meets the rest of the fluid film, and viscosity controls the length scale over which this dissipation occurs. Ultimately, viscosity only impacts the time it takes to reach this speed and not its value. The variability of the terminal speeds observed in experiments is attributed, at least partly, to changes in the local thickness of the film, the value and dynamics of which we were not able to fully resolve in numerical simulations, observations, or experiments owing to the multi-scale nature of the phenomenon and the opacity of mud.

## Data Availability

All of the data necessary to reproduce the field observations and lab experiments can be found in the Zenodo repository (3.6 GB). The Zenodo repository also contains all code necessary to reproduce figures, and the code necessary to reproduce the numerical models of bubble ascent shown in the text [61]. Supplementary material is available online [62].
